# Metal Complexation for the Rational Design of Gemcitabine Formulations in Cancer Therapy

**DOI:** 10.1021/acsami.4c12550

**Published:** 2024-10-08

**Authors:** Federica Carnamucio, Claudia Foti, Massimiliano Cordaro, Franz Saija, Giuseppe Cassone, Sandro R. P. da Rocha, Ottavia Giuffrè

**Affiliations:** † Department of Pharmaceutics and Center for Pharmaceutical Engineering and Sciences - School of Pharmacy, Virginia Commonwealth University, Richmond, Virginia 23284, United States; ‡ Dipartimento di Scienze Chimiche, Biologiche, Farmaceutiche ed Ambientali, Università di Messina, Viale F. Stagno d’Alcontres 31, 98166 Messina, Italy; § Institute for Chemical-Physical Processes National Research Council of Italy, Viale Ferdinando Stagno d’Alcontres, 37, 98158 Messina, Italy; ∥ Dipartimento di Scienze Chimiche, Biologiche, Farmaceutiche ed Ambientali, Università di Messina, Viale F. Stagno d’Alcontres 31, 98166 Messina, Italy

**Keywords:** gemcitabine, Mn^2+^, Zn^2+^, Ca^2+^, metal complexation, DFT calculation, ab initio, cancer therapy

## Abstract

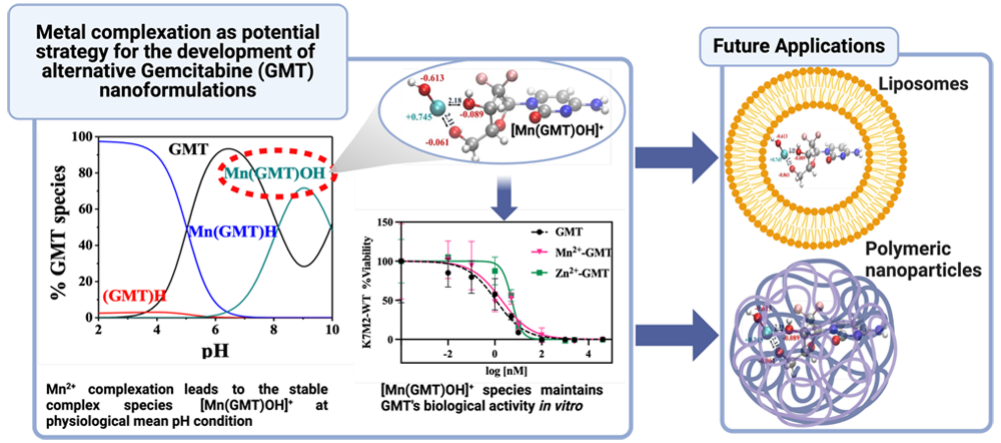

Nanoformulation of chemotherapies represents a promising strategy to enhance outcomes in cancer therapy. Gemcitabine is a chemotherapeutic agent approved by the Food and Drug Administration for the treatment of various solid tumors. Nevertheless, its therapeutic effectiveness is constrained by its poor metabolic stability and pharmacokinetic profile. Nanoformulations of gemcitabine in lipid and polymer nanocarriers usually lead to poor loading capability and an inability to effectively control its release profile due to the physicochemical characteristics of the drug and matrices. Here, we propose metal–gemcitabine complexation with biorelevant metal cations as a strategy to alter the properties of gemcitabine in a noncovalent manner, paving the way for the development of novel nanoformulations. A speciation study on gemcitabine and Mn^2+^, Zn^2+^, and Ca^2+^ was performed with the aim of investigating the extent of the interaction between the drug and the proposed metal cations, and selecting the best conditions of temperature, pH, and drug-to-metal molar ratio that optimize such interactions. Also, a series of density functional theory calculations and spin-polarized *ab initio* molecular dynamics simulations were carried out to achieve insights on the atomistic modalities of these interactions. Mn^2+^-gemcitabine species demonstrated the ability to maintain gemcitabine’s biological activity *in vitro*. The scientific relevance of this study lies in its potential to propose metal-gemcitabine as a valuable strategy for developing nanoformulations with optimized quality target product profiles. The work is also clinically relevant because it will lead to improved treatment outcomes, including enhanced efficacy and pharmacokinetics, decreased toxicity, and new clinical possibilities for this potent therapeutic molecule.

## Introduction

**Figure 1:**
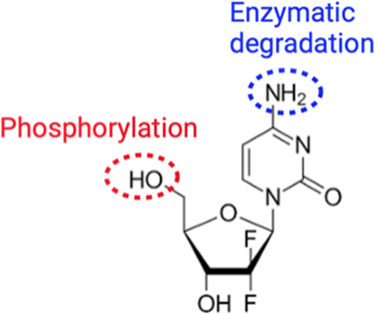
Gemcitabine (GMT) structure.

Gemcitabine or 2′-deoxy-2′,2′-difluorocytidine (GMT), whose structure is reported in Figure 1, is a cytidine analogue in which two fluorine atoms replace the hydroxyl on the ribose. GMT is intracellularly phosphorylated and converted to the active moiety dFdCDP and dFdCTP, which induce apoptosis by DNA recognition. After intravenous administration, ≥99% of GMT is rapidly deaminated to 2′,2′-difluorodeoxyuridine (dFdU) by deoxycytidine deaminase present in the blood and liver and then subjected to a fast renal clearance. GMT is approved by the Food and Drug Administration (FDA) for the treatment of various solid tumors, including pancreatic, breast, bladder, head, neck, thyroid, ovarian, nonsmall cell lung, bone, and biliary tract cancers.^1,2^ It is an essential medicine according to the World Health Organization (WHO).^3^ Administered as a prodrug, GMT is phosphorylated to form the active moiety GMT triphosphate (dFdCTP) and GMT diphosphate (dFdCDP), which inhibit DNA synthesis.^4^ Upon intravenous administration, it is rapidly deaminated by cytidine deaminase, and cleared by the kidneys.^5^ Over 99% of GMT is fully deaminated in the blood, liver, kidneys, and other tissues in the inactive metabolite 2′,2′-difluorodeoxyuridine (dFdU) and excreted by urine.^6^ While of tremendous benefit in the treatment of various types of malignancies, GMT has shown a rapid metabolism and a poor pharmacokinetic (PK) profile (plasma half-life *T*_1/2_ less than 30 min), thus limiting its potential as a chemotherapeutic agent.^7^ Due to its poor PK profile,^7^ high doses and prolonged infusion times are typically required to keep the concentrations within the therapeutic window, which may lead to myelosuppressive and other side effects.^8,9^ Nanodrug delivery systems have been proposed to improve GMT PK and biodistribution profiles, as they protect the drug against enzymatic degradation and, due to a GMT controlled release, improve PK tumor distribution.^10^

The use of nanoformulations in chemotherapies has been shown to be effective in decreasing toxicity, increasing selectivity and enhancing drug efficacy.^11−13^ Polymeric and lipid (e.g., liposomes) nanoparticles represent two prominent approaches in nanodrug delivery systems, particularly for anticancer drugs.^14^ Each approach offers distinct advantages. Liposomes, for instance, are highly biocompatible and biodegradable,^15^ whereas polymeric nanoparticles are known for their robust structural integrity, stability under storage conditions, and controlled release capabilities.^16^ In spite of their excellent carrier properties are known, GMT nanoformulations are characterized by low drug loading (DL%, amount of drug per amount of lipid formulation) and low entrapment efficiency (EE%, extent of drug that is encapsulated within the nanocarrier).^17^ Additionally, liposomes face challenges for the reduced physical stability under storage conditions.^18^ Metal cations have frequently been utilized in nanoformulations due to their ability to enhance drug loading through drug complexation.^19^ For instance, Vyxeos was approved in 2017, a multilamellar lipid formulation that uses copper complexation for enhancing loading of cytarabine (which has a similar chemical structure to GMT) and daunorubicin.^19^ Starting from these premises, the goal of this research is to study the interaction between GMT and biorelevant metal cations in order to propose metal complexation as a potential strategy for the development of new GMT nanoformulations. Mn^2+^, Zn^2+^, and Ca^2+^, commonly present in biological fluids, are proposed as candidates for their importance in many physiological activities,^20−22^ and for their ability to modulate the tumor microenvironment by inducing M1 polarization (Mn^2+^) in tumor-associated macrophages,^23^ activating the Wnt-3a/β-catenin signaling pathway (Zn^2+^) in cancer cells,^24^ and treating the hepatocarcinogenesis (Ca^2+^).^25^ Moreover, Mn^2+^, Zn^2+^, and Ca^2+^ are clinically relevant, being used as adjuvants all via systemic administration.^26−28^

First, the acid–base behavior of GMT was investigated by performing spectrophotometric titrations on GMT solutions under various temperature conditions (15, 25, 37, and 45 °C). Then, formation constants of Mn^2+^, Zn^2+^, and Ca^2+^–GMT species were obtained by using above all potentiometric, spectrophotometric, and ^1^H NMR titrations. Also, the efficacy of M^2+^–GMT (metal–GMT) vs free GMT and free M^2+^ in promoting cell death was tested *in vitro* in a murine osteosarcoma cell line (K7M2). Furthermore, a series of computational investigations were carried out by using traditional density functional theory calculations under implicit solvation as well as via state-of-the-art spin-polarized *ab initio* molecular dynamics simulations with explicitly treated solvent molecules at a finite temperature to investigate the molecular modalities of interaction of Mn^2+^–GMT species as well as the structure of the liquid environment around GMT and Mn^2+^–GMT species. The reported results led to the identification of optimal conditions for pH, temperature, and the drug-to-metal molar ratio that maximize M^2+^–GMT interactions. These findings will pave the way for GMT nanoformulations with desirable quality attributes for the treatment of various cancers.

## Materials and Methods

### Materials

Manganese, zinc, and calcium solutions were obtained by weighing and dissolving the corresponding salt manganese(II) sulfate monohydrate (Sigma-Aldrich, purity >99%), zinc(II) chloride eptahydrate (Sigma-Aldrich, puriss.) and calcium(II) chloride dihydrate (Fluka, purity >99%). All metal cation solutions were standardized by titration with EDTA (ethylenediamine tetraacetic acid disodium salt, Sigma-Aldrich, purity ≥99%) standard solution. GMT solutions were prepared by weighing and dissolving GMT hydrochloride (TCI, purity >98%). Solutions of hydrochloric acid and sodium hydroxide were obtained by dilution of Fluka ampules, subsequently standardized with dried sodium carbonate (Sigma-Aldrich, purity ≥99.5%), and potassium biphthalate (Sigma-Aldrich, purity ≥99.5%), respectively. Solutions of sodium hydroxide were frequently prepared and stored in bottles with soda lime traps. Solutions of sodium chloride were obtained by weighing the corresponding salt (Sigma-Aldrich, puriss.), previously dried in an oven at 110 °C. Distilled water (conductivity <0.1 μS cm^–1^) and grade A glassware were employed for the preparation of all the solutions. Phosphate buffer saline (PBS, 1X), Dulbecco’s Modified Eagle’s Medium (DMEM), and trypsin-EDTA solution (0.25% trypsin and 0.53 mM EDTA) were purchased from Life Technologies. Fetal bovine serum (FBS) was purchased from Atlanta Biologicals (Flowery Branch, GA). Amicon Ultra-4 centrifugal filter device (MWCO = 10 kDa) was purchased from EMD Millipore (Billerica, MA). Tissue culture flasks (T25 and T75) were purchased from VWR International (Radnor, PA). Round bottom 96-well plates (Cellstar) were purchased from Greiner Bio-One (Kremsmünster, Austria).

### Potentiometric Apparatus and Procedure

Two distinct systems were employed for the potentiometric titrations, both arranged in an identical configuration of an automatic dispenser Metrohm Dosino 800, a Metrohm model 809 Titrando potentiometer, and a Metrohm LL-Unitrode WOC combined glass electrode. Each potentiometric system was connected to a PC, and the experimental titration data were acquired by Metrohm TIAMO 2.2 software. The estimated accuracy of both systems is ±0.15 mV for the emf and ±0.002 mL for the titrant volume. Each titration consists in additions of volumes of NaOH standard to 25 mL of the solution containing GMT, HCl and a supporting electrolyte (NaCl) for the acid–base study and M^2+^ salt (M^2+^ = Mn^2+^ or Zn^2+^ or Ca^2+^), GMT, HCl and NaCl for the complexation study. Experimental details on the potentiometric titrations are reported in Table 1. For all measurements, glass jacket thermostated cells were used to perform titrations under different temperatures (15 ≤ *t*/°C ≤ 45). In addition, pure N_2_ was bubbled to remove CO_2_ and O_2_. For each measurement, an independent titration of HCl with standard NaOH was performed to calculate the standard electrode potential E^0^ and the p*K*_w_ value under the same experimental ionic strength and temperature.

**Table 1:** Experimental Conditions for Potentiometric, Spectrophotometric, and NMR Titrations (pH Range 2–10)

technique	*t* °C	*C*_M_^a^ mmol L^–1^	*C*_GMT_^b^ mmol L^–1^	M/GMT ratio
potentiometry	15, 25, 37, 45	0.5	0.5	1:1
		0.5	0.75	1:1.5
		0.5	1	1:2
		1	0.5	2:1
spectrophotometry	25, 37, 45		0.04–0.08	
	45	0.02	0.04	1:2
	45	0.035	0.05	1:1.4
	45	0.02	0.08	1:4
	45	0.04	0.04	1:111
^1^H NMR	25		5	
	25	6	5	1.2:1

a Metal cation concentration.

b GMT concentration.

### UV–Vis Apparatus and Procedure

The spectrophotometric titrations were carried out by a Varian Cary 50 UV–vis spectrophotometer equipped with an optical fiber with a fixed 1 cm path length, interfaced to a PC by the Varian Cary WinUV software, and an automatic dispenser Metrohm 715 Dosimat. For each titration point, the couple data of absorbance (Abs) and pH vs volume of titrant (mL) were recorded simultaneously by using a Metrohm glass electrode and a Metrohm-713 pH meter. All titrations were performed using a thermostated cell to keep the desired temperature under N_2_ to remove CO_2_ and O_2_ from the solutions. The experimental conditions of the titrations are listed in Table 1. For each system, at least four measurements were performed, scanning the spectral range 225 nm ≤ λ ≤ 350 nm in different conditions of GMT concentration and metal–GMT (M/GMT) ratio. Before each measurement, a baseline containing only HCl, NaCl, and H_2_O was recorded to subtract the matrix contribution.^11^

### ^1^H NMR Apparatus and Procedure

The spectrometer employed for the collection of ^1^H NMR spectra was a Varian 500 FT-NMR. 1,4-dioxane was used as internal reference (δ_CHdioxane_ = 3.70 ppm), and the chemical shifts are referred to the tetramethylsilane (TMS). All the measurements were carried out in a 9:1 H_2_O/D_2_O solution of GMT or Zn^2+^–GMT at *t* = 25 °C, suppressing the water signal by the presaturation technique. Experimental details on NMR titrations are reported in Table 1.

### Cell Culture

10^4^ cells/mL of K7M2-WT were plated in 25 cm^2^ cell culture flasks and cultured by DMEM with 10% FBS and 1% PS. The cells were grown in a Thermo Scientific CO_2_ incubator at 37 °C and 5% CO_2_. The medium was exchanged every 2/3 days. The cells were split as they reached ca. 80–90% of confluency.

### K7M2-WT Cell Viability

K7M2-WT cells were seeded in 96-well plates at a density of 10^4^ cells per well. The cells were allowed to grow (DMEM + 10% FBS + 1% PS) for 24 h at 37 °C and 5% CO_2_. The drug solution was added in each well at different GMT or GMT-equivalent concentrations between 0.01 and 2500 nmol L^–1^. After the treatment, the cells were incubated for an additional 48 h under 5% CO_2_ at 37 °C in DMEM + 10% FBS + 1% PS. Cell viability was analyzed using MTT (3-(4, 5-dimethylthiazolyl-2)-2, 5-diphenyltetrazolium bromide) assay (Invitrogen, Thermo-Fisher Scientific). Briefly, the cells were rinsed twice with PBS and 110 μL of 1.09 mmol L^–1^ MTT (in PBS) was added to each well, followed by plate incubation for 4 h at 37 °C and 5% CO_2_. Subsequently, 80 μL of solution was removed from each well and 55 μL of DMSO (dimethylsulfoxide) was added and mixed. The plates were, then, covered and incubated at 37 °C for an additional 10 min. The absorbance was read at 540 nm using a multimode microplate reader (Synergy H1, Biotek). Cell viability was calculated as follows.
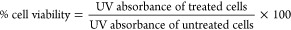


### Postprocessing Calculations

Protonation constant values, speciation model for each metal-complex system, formation constant values, standard potential *E*^0^, analytical concentration of the reagents, and junction potential of each titration were determined by processing potentiometric results by the BSTAC4 and STACO4 programs. The parameters for the dependence of complex formation constants on temperature were obtained using LIANA. More details on the software employed in the refinement of the experimental data are reported in ref (29). For ^1^H NMR titrations, the HypNMR software was employed to obtain protonation and formation constant values, as well as the individual chemical shift of each species, using the observed signals and assuming fast mutual exchange in the NMR time scale.^30^ HypSpec program was used for UV data, enabling us to refine protonation and formation constants as well as the molar absorption coefficient values (ε) of all species. The HySS program was used to obtain the speciation diagrams and the formation percentages of the complex species.^31^

### Density Functional Theory Calculations and *Ab Initio* Molecular Dynamics

All static calculations were performed by means of the Gaussian 16 software.^32^ DFT was used to evaluate the ground-state structures of the selected molecular species. In this work, the B3LYP^33^ hybrid exchange and correlation functional, with 100% of exact exchange, was employed. Geometry optimizations of the molecular structures were performed under implicit solvation conditions using the 6-311++G(d,p) atomic basis set for all of the atoms. In addition, when the manganese cation was included, the Stuttgart–Dresden (SDD) basis set was employed for this species. The latter basis set treats the inner core electrons with a constant pseudopotential, and the valence electrons with a triple-ζ valence basis set. We employed this strategy to enhance the computational efficiency when describing transition metals. The conductive polarizable continuum model (CPCM)^34^ with typical parameters was employed for water.

Whereas GMT is neutral and typically found in a singlet electronic state, when GMT binds the [MnOH]^+^ cation, the formed complex is an open-shell species with a global positive charge of +1 lying in a doublet spin state. Unrestricted spin-polarized DFT calculations were performed in those cases. Born–Oppenheimer AIMD simulations were performed by using the CP2K^35^ software suite on two different numerical samples. The first one contained one GMT and 142 H_2_O molecules (*i.e*., 455 atoms), whereas the second one was composed of one GMT+[MnOH]^+^ complex surrounded by 143 water molecules (*i.e*., 461 atoms). While in the former case, a neutral simulation box with a global spin component equal to one was simulated, a simulation box having a global charge equal to +1 and a spin component of 2 was imposed in a spin-polarized AIMD simulation. Initial structures of GMT and GMT+[MnOH]^+^ were preoptimized at the B3LYP/6-311++G(d,p) level with the CPCM water as an implicit solvation model. As for the GMT+[MnOH]^+^, in light of the observed higher stability with respect to other plausible molecular arrangements, only the complex with the [MnOH]^+^ moiety chelated between the two hydroxyl groups of GMT was simulated *via* spin-polarized AIMD. Cubic supercells with edges of 16.22 and 16.85 Å were built for the systems containing the neutral GMT structure and the GMT+[MnOH]^+^ complex, respectively. Periodic boundary conditions were applied along the three Cartesian axes to mitigate spurious finite-size effects. During AIMD simulations, wave functions of each atomic species were expanded in double-zeta-valence-plus-polarization (DZVP) basis sets with the Goedecker–Teter–Hutter pseudopotentials^36^ using the GPW method.^37,38^ A plane-wave cutoff of 400 Ry was adopted. Exchange and correlation effects were treated *via* the PBE^39^ density functional, belonging to the Generalized Gradient Approximation (GGA) DFT class. Moreover, to take into account dispersion interactions, the dispersion-corrected PBE+D3^40,41^ functional was employed. Each trajectory was simulated for 15 ps at a nominal temperature of 330 K. A temperature higher than the room one was selected to mitigate the well-known overstructuring of the solvation environment introduced by GGA DFT functionals in liquid water and for more closely mimicking the operational conditions of the experiments. The temperature was kept fixed via a CSVR^42^ thermostat set with a time constant equal to 50 fs. In this way, the systems were simulated in an isothermal-isochoric (NVT) ensemble, whereas the nuclei dynamics were classically propagated through the Verlet algorithm with a time-step of 0.5 fs.

## Results and Discussion

### Acid–Base Behavior

A preliminary acid–base study of GMT was performed to assess its protonation constant values, which were needed for the subsequent metal–ligand investigation. The values of the protonation constant of the amine group in the 4′ position in GMT, determined at different temperatures (15, 25, 37, and 45 °C), are listed in Table 2. To the best of our knowledge, the only related data present in the literature refers to GMT’s log *K* = 3.60 at *t* = 25 °C and unspecified ionic strength.^43^ This value coincides with that at *t* = 25 °C and *I* = 0.1 mol L^–1^ determined by us and reported in the current study.

**Table 2:** Experimental Protonation and Formation Constants of H^+^–GMT, Zn^2+^–GMT, Mn^2+^–GMT, and Ca^2+^–GMT Species at *I* = 0.1 mol L^–1^

reaction	*t* /°C	log β ± SD^a^
	15	3.94 ± 0.03
	25	3.60 ± 0.02
	37	3.51 ± 0.02
	45	3.55 ± 0.02
	25	7.64 ± 0.04
	37	7.55 ± 0.04
	45	7.63 ± 0.06
	25	–5.00 ± 0.04
	37	–4.73 ± 0.04
	45	–4.40 ± 0.03
	25	6.39 ± 0.03
	37	6.50 ± 0.01
	45	7.24 ± 0.08
	25	–6.52 ± 0.02
	37	–6.15 ± 0.07
	45	–5.55 ± 0.08
	25	6.79 ± 0.05
	37	6.64 ± 0.09
	45	6.38 ± 0.03

reaction	*t* /°C	log *K*
	25	4.04
	37	4.04
	45	4.08
	25	4.14
	37	4.05
	45	4.38
	25	2.79
	37	2.99
	45	3.69
	25	3.94
	37	3.05
	45	4.16
	25	3.19
	37	3.13
	45	2.83

a SD = standard deviation.

**Figure 2:**
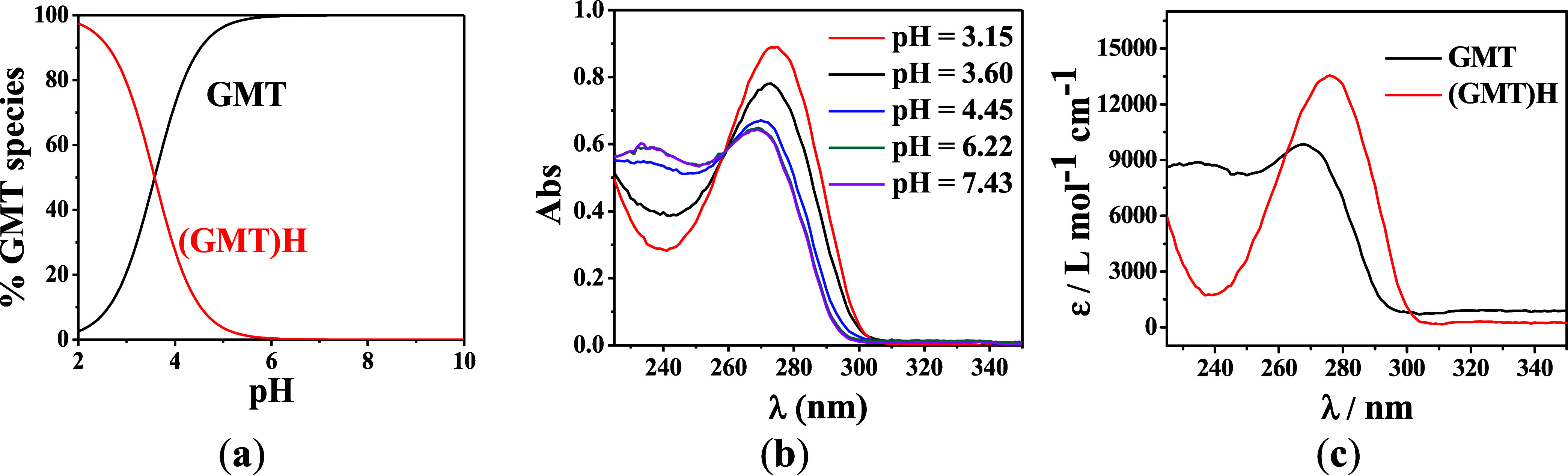
(a) Speciation diagram of GMT species; (b) UV spectra of GMT solution at selected pH values at *C*_GMT_ = 0.06 mmol L^–1^; (c) molar extinction coefficients (ε) vs wavelength (λ). Experimental conditions: *t* = 37 °C, *I* = 0.1 mol L^–1^ in NaCl.

The speciation diagram of GMT obtained at *t* = 37 °C and *I* = 0.1 mol L^–1^ is reported in Figure 2a. The diagram shows that at pH = 2.0, almost 100% of GMT is protonated. At pH = 6.0, all of the ligand is deprotonated. UV spectra obtained at selected pH are reported in Figure 2b. A maximum of absorption is distinguishable at λ = 270 nm, which shows a blueshift in the spectrum as a consequence of pH increase. From pH = 3.15 to 6.22, a hypochromic effect is observed, but no changes are found in the pH range 6.22 ≤ pH ≤ 7.43. An isosbestic point at λ = 260 nm, representing GMT/[(GMT)H]^+^ equilibrium, is also evident. The molar extinction coefficients (ε) vs. wavelength (λ) of GMT species are reported in Figure 2c. More in detail, two maxima of 14 000 L mol^–1^ cm^–1^ at λ = 280 nm and 9 500 L mol^–1^ cm^–1^ at λ = 275 nm are observed for [(GMT)H]^+^ and GMT species, respectively.

### Mn^2+^–GMT, Zn^2+^–GMT, and Ca^2+^–GMT Complexes

The study of the interaction between GMT and Mn^2+^, Zn^2+^, and Ca^2+^ was performed with the aim to use metal complexation for altering the physiochemical properties of GMT leading to the development of enhanced GMT nanoformulations in lipid or polymeric matrices. Once the acid–base properties of the ligand as well as the hydrolytic behavior of the metal cations (hydrolytic constants reported in Tables S1–S3 of Supporting Information) were determined, we then investigated the interaction between GMT and metal cations. With the aim of choosing the most reliable speciation model, potentiometric titrations were performed on solutions with different metal–ligand ratios.

All the experimental formation constant values of the GMT complex species under different temperature conditions are reported in Table 2. To have an immediate comparison between the stability of the different species, the partial formation constants (log *K*) were also calculated and are reported in Table 2. All the protonation and formation constant values, listed in Table 2, were calculated by fitting the experimental potentiometric data with BSTAC4 and STACO4 programs.

**Figure 3:**
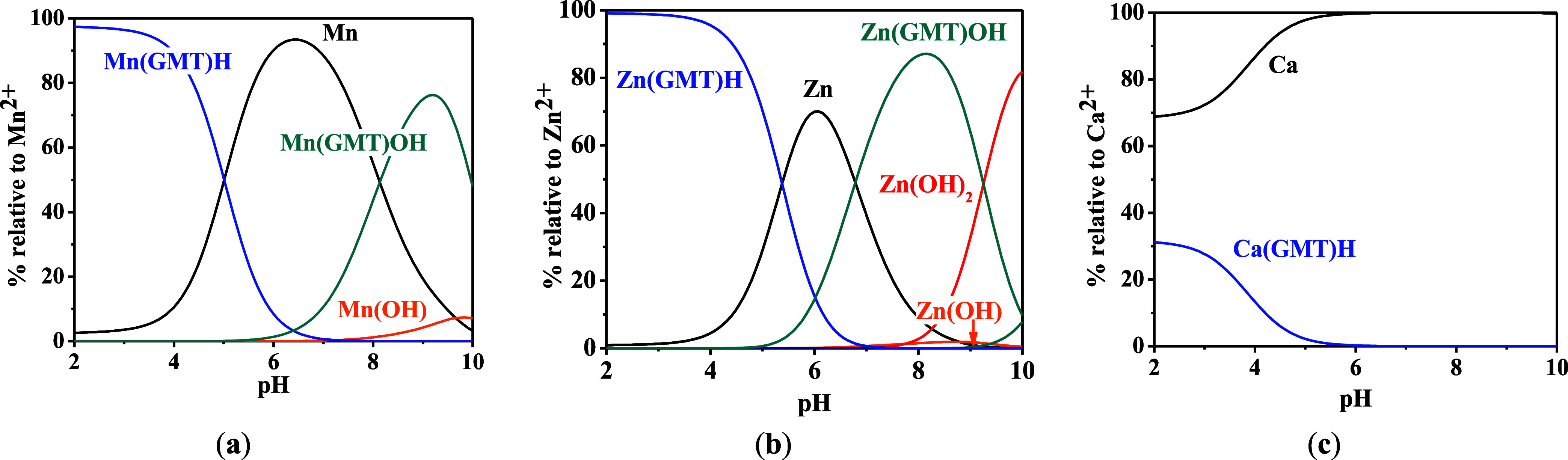
Distribution diagrams of (a) Mn^2+^–GMT, (b) Zn^2+^–GMT and (c) Ca^2+^–GMT systems at *t* = 45°, *C*_GMT_ = *C*_M_ = 1 mmol L^–1^ and *I* = 0.1 mol L^–1^.

For all the systems, the most reliable speciation model was selected by doing several trials on potentiometric titrations with different metal–ligand ratios, testing different models, and choosing the one that met criteria such as simplicity, lowest mean deviation parameters, and higher formation percentages of the species, as indicated in the ref (44). More in detail, for Mn^2+^–GMT and Zn^2+^–GMT the same speciation pattern was found, which includes [M(GMT)H]^3+^ (resulting from the protonation of GMT and subsequent interaction with the free metal cations) and [M(GMT)OH]^+^ species (formed by the interaction of the hydrolytic species of the metal cations and free GMT). A simpler speciation model has emerged for Ca^2+^–GMT, with the latter showing the formation of the [Ca(GMT)H]^3+^ species only. As shown in Figure 3a, at *t* = 45 °C, the [Mn(GMT)H]^3+^ species, found in the range 2 ≤ pH ≤ 6, reaches its maximum formation percentage, corresponding to 98% at pH = 2. [Mn(GMT)OH]^+^ species, formed in the range 7 ≤ pH ≤ 10, reaches 70% at pH = 9. This complex formation reduces the [MnOH]^+^ percentage in solution, which reaches a maximum of 10% at pH 10. Figure 3b shows the speciation of the Zn^2+^–GMT system at *t* = 45 °C, where the [Zn(GMT)H]^3+^ complex exists in the range 2 ≤ pH ≤ 6, reaching a maximum of 98% at pH = 2. On the other hand, the [Zn(GMT)OH]^+^ species forms at 5.5 ≤ pH ≤ 10, with a maximum fraction equal to 95% at pH = 8. The hydrolytic species [ZnOH]^+^, due to the formation of a high amount of the complex species, reaches a maximum of 2% at pH = 8.5. As displayed in Figure 3c, [Ca(GMT)H]^3+^ species is formed in the range 2 ≤ pH ≤ 5 with a maximum of 31% at pH = 2 at *t* = 45 °C. The weak hydrolytic species [CaOH]^+^ does not form a significant percentage in the investigated pH range, so it is not present in the reported speciation diagram. By comparing the log *K* value reported in Table 2, both Mn^2+^–GMT and Zn^2+^–GMT systems exhibit a higher stability at *t* = 45 °C than *t* = 37 °C or *t* = 25 °C. Conversely, the Ca^2+^–GMT system shows better stability at physiological temperature conditions (37 °C). Moreover, according to these preliminary results, Mn^2+^ and Zn^2+^ interactions lead to more stable complex species than Ca^2+^, as already observed in several other studied systems.^45,46^ Also, *t* = 45 °C, 1:1 = M/GMT molar ratio, and pH = 7.4 (mean physiological pH) for Mn^2+^ and Zn^2+^, may be suitable to prepare nanoformulations as we can achieve higher formation percentages of metal complexes under those conditions.

The two systems Mn^2+^–GMT and Zn^2+^–GMT were also investigated by spectrophotometric titrations to confirm the formation constant values and the speciation models and to examine the spectral behavior of the complex species. A comparison between the formation constants determined by potentiometry and spectrophotometry at *t* = 45 °C is reported in Table 3. The formation constant values obtained by spectrophotometry agree with those obtained by potentiometry.

**Table 3:** Comparison between Experimental Formation Constants Obtained by Potentiometry, UV Spectrophotometry, and ^1^H NMR Spectroscopy at *t* = 45°C and *I* = 0.1 mol L^–1^

reaction	log β_UV_ ± SD^a^	log β_potentiometry_ ± SD^a^	log β_NMR_ ± SD^a^
	7.63 ± 0.07	7.63 ± 0.06	7.63 ± 0.08
	–4.49 ± 0.04	–4.40 ± 0.03	–4.24 ± 0.08
	7.36 ± 0.08	7.24 ± 0.08	
	–5.62 ± 0.09	–5.55 ± 0.08	

a SD = standard deviation.

**Figure 4:**
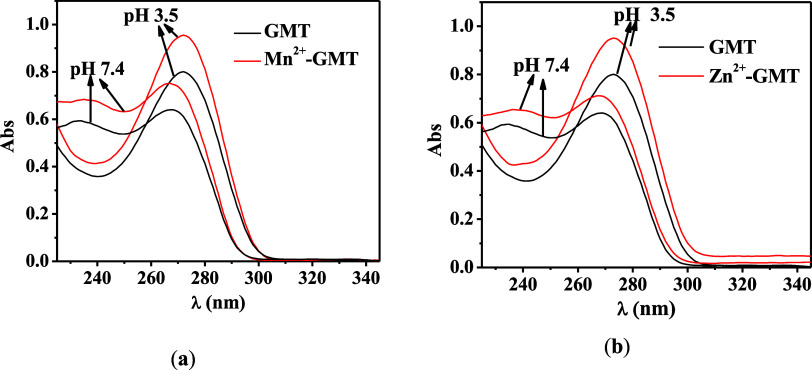
UV spectra at selected pH of (a) Mn^2+^–GMT and (b) Zn^2+^–GMT at *t* = 45 °C, *I* = 0.1 mol L^–1^, and *C*_GMT_ = *C*_M_ = 0.04 mmol L^–1^.

Figure 4 displays the UV spectra for free GMT and M^2+^–GMT systems at pH values of 3.5 and 7.4. For both systems, a hyperchromic effect is observed at these pH values, indicating the formation of a complex species. Specifically, (a) the absorbance change (ΔAbs) is 0.14 at λ = 270 nm and pH 3.5, and ΔAbs = 0.09 at λ = 280 nm and pH 7.4; (b) ΔAbs is 0.12 at λ = 270 nm and pH 3.5, and ΔAbs = 0.07 at λ = 280 nm and pH 7.4.

In Figure S2 (Supporting Information), some of the spectra acquired on solutions containing Mn^2+^–GMT and Zn^2+^–GMT at the selected pH are reported.

**Figure 5:**
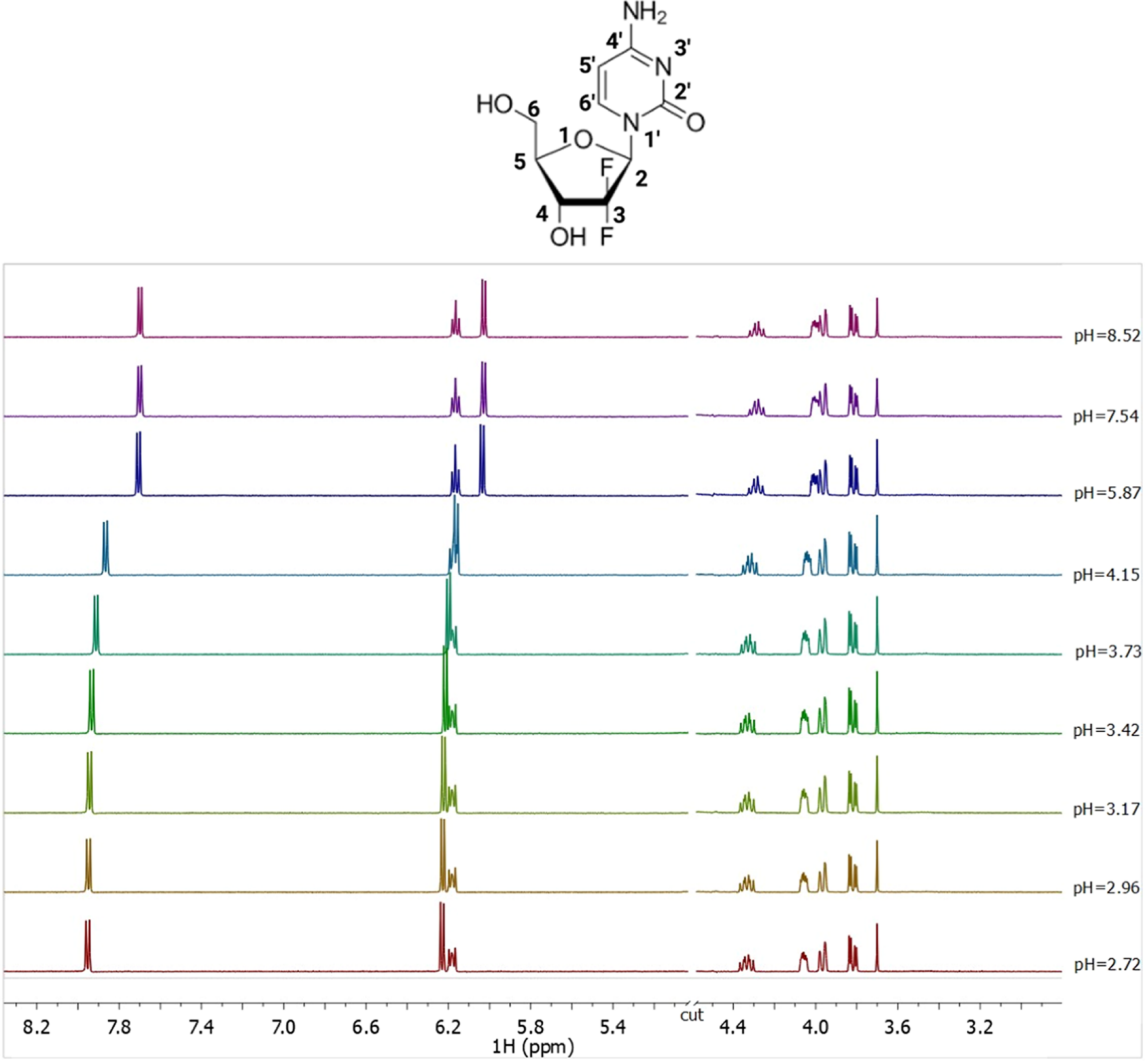
^1^H NMR spectra on solutions containing GMT at *C* = 5 mmol L^–1^, *t* = 25 °C, *I* = 0.1 mol L^–1^ in NaCl, and 2.72 ≤ pH ≤ 8.52.

As far as we know, there are no stability data in the literature regarding the formation of gemcitabine complexes with Ca^2+^, Mn^2+^, or Zn^2+^ metal cations. However, it is possible to consider cytidine, as the gemcitabine molecule is a deoxycytidine where the fluorine atoms replaced the hydrogen atoms on the 3 carbon (see Figure 5). In the case of cytidine, there are some papers in the literature, albeit dated, on the complexation with Mn^2+^ and Zn^2+^^47,48^ Reddy and Rao report the formation constants of the species at *t* = 35 °C and *I* = 0.1 mol L^–1^ in KNO_3_. For Mn^2+^-cytidine and for Zn^2+^-cytidine the speciation models include only the ML species with stability constants equal to 2.51 and 2.60, for Mn^2+^ and Zn^2+^, respectively.^47^ Although the speciation models and ionic media differ and GMT and cytidine are not identical, the stability of the species remains comparable, particularly for Mn^2+^. For example, log*K* for [Mn(GMT)H]^3+^ species is 2.99 at *I* = 0.1 mol L^–1^ in NaCl and *t* = 37 °C (see Table 2). In the literature, referring to cytidine-5′-triphosphoric acid, a paper reports the IUPAC evaluation on stability data of species formed by this ligand with Mn^2+^ and Zn^2+^ at *t* = 25 °C and *I* = 0.1 mol L^–1^ in R_4_NX.^48^ The speciation model for both Mn^2+^ and Zn^2+^ includes two species, namely, ML and MLH. The log*K* values referring to the species common to the literature model and ours, that is MLH, are 3.0 and 2.88 for Mn^2+^ and Zn^2+^, respectively. The value here reported for the gemcitabine species with Mn^2+^, having the same stoichiometry and the same formation reaction, under the same temperature and ionic strength in a different ionic medium (NaCl), is log*K* = 2.79. This value is fairly close to that of cytidine-5′-triphosphoric acid.

Several ^1^H NMR titrations were performed on GMT solutions. All ^1^H NMR spectra at selected pH values and *t* = 25 °C show seven signals, as displayed in Figure 5. Two double doublets at 3.8 and 3.9 ppm related to CH_2_ protons, two multiplets at 4.1 and 4.3 ppm assigned to the CH-4 and CH-5, respectively, a multiplet at 6.1 ppm of the CH-2, and two doublets at 6.2 and 7.9 ppm related to the CH-5′ and CH-6′ protons. By increasing the pH value, an upfield of 0.2 ppm of the CH-5′ and CH-6’ protons chemical shift can be observed due to the deprotonation of the amine group of the pyrimidine ring.

The Zn^2+^–GMT system was also investigated via ^1^H NMR titrations. The same trend of free GMT was found for the Zn^2+^–GMT system (Figure S3 in Supporting Information). The analysis of the NMR experimental data confirms the speciation model of the Zn^2+^–GMT system and allows to obtain the formation constant values of both species, despite the chemical shift differences referred to GMT and GMT with Zn^2+^ are very small (for CH-5′ and CH-6′ protons Figure S4 in Supporting Information). This is often observed for weak interactions in aqueous solutions and when the species, involved in the equilibria, are rapidly exchanging on the NMR time scale. The formation constant values, reported in Table 3, obtained via ^1^H NMR spectroscopy closely match those gathered via potentiometry and UV spectrophotometry (no statistical differences have been found, *p* = 0.8333, One-way ANOVA). By the determination of the protonation and formation constants under different temperatures, listed in Table 2, it is possible to calculate the protonation and formation enthalpy change values. More in detail, the protonation and formation constants obtained by the potentiometric titrations under various temperatures (calculated by BSTAC4 and STACO4 programs), were processed using the LIANA fitting program, and taking into account the following van’t Hoff equation^49^
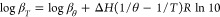


where log β_T_ is the stability constant at a certain temperature (expressed in Kelvin), log β_θ_ is the stability constant at *T* = 298.15 K, and *R* is the universal gas constant expressed as 8.314 J K^–1^ mol^–1^. The values of protonation and formation enthalpy changes of GMT and M^2+^–GMT species are collected in Table 4, together with the entropy and free energy values. All formation reactions exhibit negative changes in free energy, indicating that they can occur spontaneously

**Table 4:** Δ*G*, Δ*H*, and *T*Δ*S* of GMT, Zn^2+^–GMT, Mn^2+^–GMT, and Ca^2+^–GMT Species at *t* = 25°C and *I* = 0.1 mol L^–1^ in NaCl

reaction	Δ*G*^a^	Δ*H* ± SD^a^^,^^b^	*T*Δ*S*^a^
	–20.5	–20 ± 8	1
	–23.1	22 ± 5	45
	–23.6	44 ± 5	68
	–15.9	88 ± 7	104
	–22.5	10 ± 6	32
	–18.2	–11 ± 3	7

a In kJ mol^–1^.

b SD = standard deviation.

### *In Vitro* Evaluation Activity

**Figure 6:**
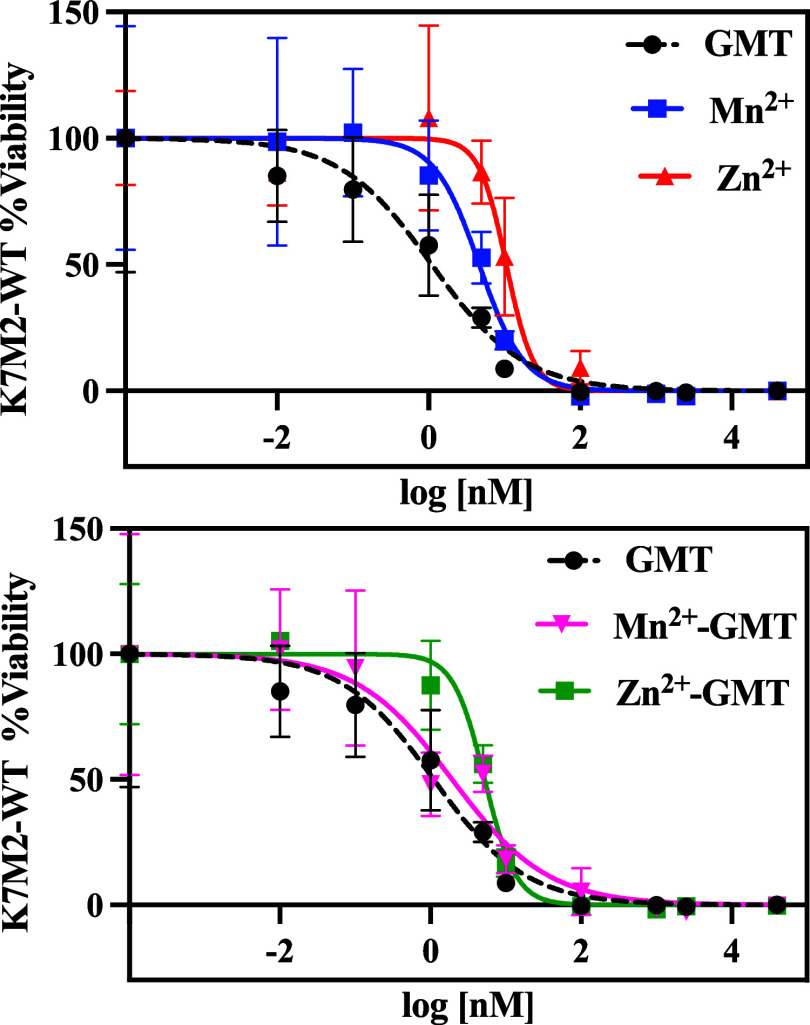
Viability of K7M2-WT at 48 h after exposure to therapy. IC_50_: GMT 1 nM (95% CI of 1 to 2 nM); Mn^2+^ 5 nM (95% CI of 3 to 7 nM), Zn^2+^ 11 nM (95% CI of 8 to 20 nM), Mn^2+^–GMT 1 nM (95% CI of 1 to 4 nM), and Zn^2+^–GMT: 5 nM (95% CI of 4 to 6 nM). Data were calculated using a nonlinear regression log(inhibitor) vs response (variable slope). *n* = 4/concentration.

GMT is known for its efficacy in treating various cancers either alone or in combination with other drugs,^3^ thus representing a promising therapeutic agent. To assess the biological activity of M^2+^–GMT complex species, specifically [Mn(GMT)OH]^+^ and [Zn(GMT)OH]^+^, the cell kill ability was investigated *in vitro* on K7M2-WT cells, an established model of murine osteosarcoma. Ca^2+^–GMT system’s toxicity was not investigated as, based on our speciation study, it did not show any complex species under physiological pH conditions (pH = 7.4). These investigations aimed to determine whether the cytotoxic effect observed with free GMT is preserved after complexation. The results, depicted in Figure 6, illustrate that both Mn^2+^ and Zn^2+^ exhibit toxicity against K7M2-WT cells, with different potency (Mn^2+^ > Zn^2+^). Moreover, Mn^2+^–GMT displays an IC_50_ value of 1 nM, as for free GMT (1 nM), suggesting that the complexation with Mn^2+^ did not alter GMT’s biological activity. Conversely, Zn^2+^–GMT exhibits reduced cytotoxicity with an IC_50_ value of 5 nM, indicating a potency decrease of at least 5-fold compared to that of the free drug. At physiological pH, the concentration of [Zn(GMT)OH]^+^ species is higher compared to [Mn(GMT)OH]^+^, resulting in a lower amount of free GMT and a reduced amount of free metal cation (25% in the Zn^2+^–GMT versus 45% in Mn^2+^–GMT, at *t* = 37 °C). Consequently, this reduction in activity is likely attributable to the lower biological activity of Zn^2+^ compared to Mn^2+^ and/or the reduced availability of free GMT and free metal cation. These findings highlight the critical role of the metal ion selection in maintaining or altering GMT’s therapeutic efficacy. We are currently evaluating the *in vivo* antitumor efficacy of M^2+^–GMT complexes for further elucidating their potential as novel cancer therapies. These efforts will aim to translate the promising cytotoxicity results observed *in vitro* into effective treatments for cancer patients.

### Density Functional Theory Calculations and *Ab Initio* Molecular Dynamics

**Figure 7:**
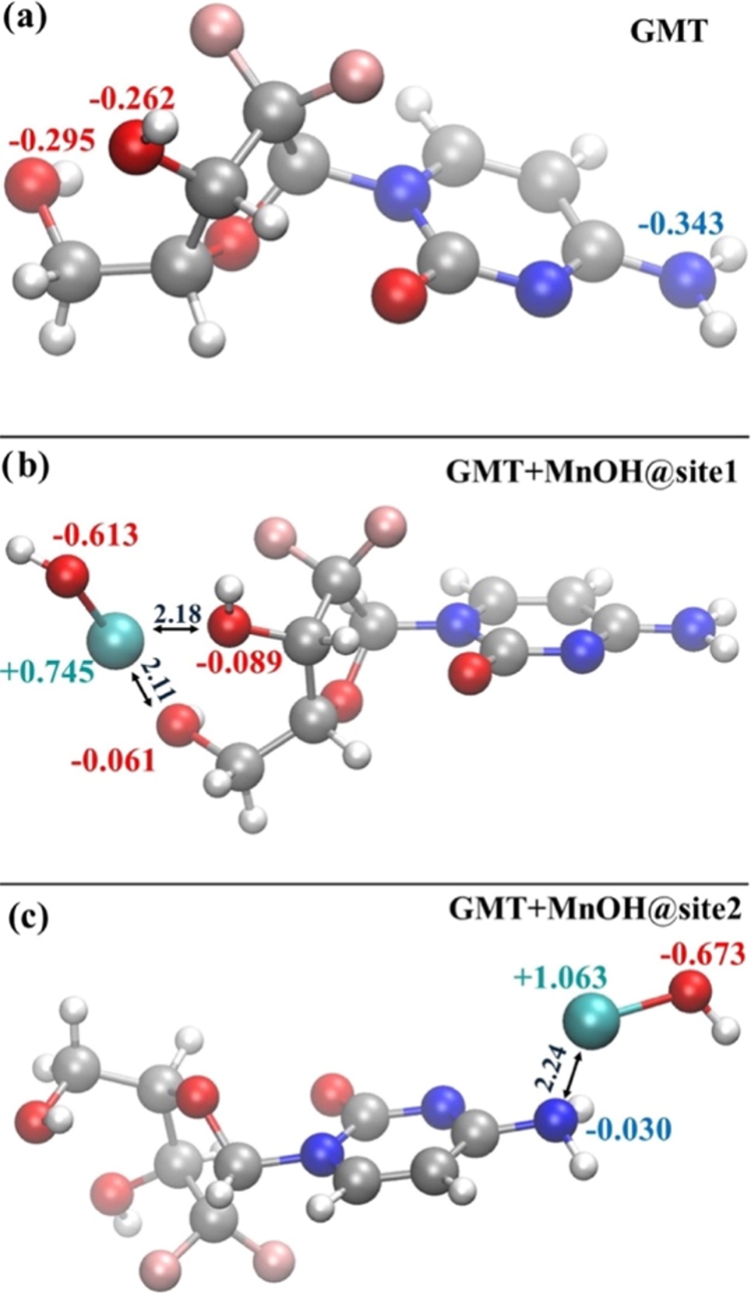
Molecular structure optimized at the B3LYP/6-311++G(d,p) level of theory under CPCM water implicit solvation of GMT (a), GMT with [MnOH]^+^ chelated by the two hydroxyl groups (b) and GMT with [MnOH]^+^ bound at the amine group (c). Colored values are the Mulliken partial charges associated with the closest atom of the same color, whereas numbers in black refer to the distance displayed by the arrows in Å.

The [Mn(GMT)OH]^+^ complex species, which exhibited higher efficacy *in vitro*, was thoroughly investigated not only by means of traditional DFT calculations but also by invoking state-of-the-art spin-polarized AIMD simulations where the active role of the solvation is explicitly and dynamically considered. To benchmark the quality of the adopted DFT framework, we report in Figure 7a the molecular structure of neat GMT optimized at the B3LYP/6-311++G(d,p) level of theory in the presence of the model solvent CPCM mimicking the surrounding water environment. The structure significantly deviates from its gas-phase ground-state configuration. Specifically, under gas-phase conditions using the B3LYP/6-311++G(d,p) level of theory, an internal hydrogen bond forms between the hydroxyl group of the ribose ring and the nearest oxygen atom.^50^ However, upon inclusion of implicit water solvent, such an internal bond disappears (Figure 7a). Despite the absence of this stabilizing intramolecular interaction, the structure’s ground-state energy under implicit solvation (Figure 7a) is lower than its gas-phase counterpart by approximately 12.2 kcal/mol (i.e., −1014.740293 vs −1014.720866 a.u).^50^ Furthermore, the lack of the internal hydrogen bond results in a less asymmetric distribution of partial charges on the oxygen atoms of the hydroxyl groups in GMT. In the presence of the CPCM solvent model (Figure 7a), the absolute charge difference is |−0.295 + 0.262| = 0.33e^–^. In contrast, this difference nearly doubles under gas-phase conditions, totaling |−0.238 + 0.182| = 0.56e^–^.^50^ Another computational study,^51^ using the more accurate M06 exchange and correlation functional and a smaller basis set (6-311G) under implicit solvation, reports a symmetric distribution of charges around these oxygen atoms, with a minimal absolute difference of |−0.276 + 0.272| = 0.006e^–^. Similar small discrepancies are noted for the nitrogen atom of the amine group: our B3LYP/6-311++G(d,p) calculations with CPCM solvation yield a partial charge of −0.343e^–^ (Figure 7a), whereas other studies report local Mulliken charges of −0.298^50^ and −0.390e^–^^51^ at the same level of theory (B3LYP/6-311++G(d,p))^50^ and under solvation with different methods (M06/6-311G with PCM).^51^ These findings show that the qualitative behavior of the electron density appears to be consistent over different DFT approaches and basis sets but preliminarily highlights important differences introduced by the inclusion, even though in an implicit manner, of the solvation environment in determining the molecular structure of GMT.

The speciation curves reported in Figure 3a, show that the GMT+[MnOH]^+^ complex, i.e., [Mn(GMT)OH]^+^, forms starting from pH ≈ 6. To investigate its molecular structure, we executed a series of unrestricted B3LYP/6-311++G(d,p) calculations under implicit solvation conditions. The two molecular sites of GMT at which the [MnOH]^+^ species can *a priori* be chemisorbed are (i) in the region between the two hydroxyl groups (site1) and (ii) in the proximity of the amine group of the pyrimidine ring (site2), whose ground-state structures are reported in Figure 7b,c, respectively. During the chelation process of [MnOH]^+^ at the site1 most of the negative charge characterizing the oxygen atoms of the hydroxyl groups of GMT is transferred to the oxygen atom of [MnOH]^+^ and, at least in part, also to the manganese atom, which exhibits a less positive local charge than expected (+0.745e^–^), as shown in Figure 7b. By contrast, a significantly more positive electric charge lies on manganese (+1.063e^–^) when the [MnOH]^+^ moiety binds at the site2, as displayed in Figure 7c. This is mainly because a larger fraction of the local electron density can be donated by the two oxygen atoms of the GMT hydroxyl groups with respect to that available around the nitrogen atom of the GMT amine group. This leads to the formation of (two) shorter bonds of 2.11 and 2.18 Å between Mn and the O atoms when [MnOH]^+^ is chelated at site1 (Figure 7b) with respect to the minimum-energy distance of 2.24 Å featuring the bond formed between Mn and N at site2 (Figure 7c). All this evidence indicates that stronger interactions arise at the site1 than at the site2. In fact, the GMT+[MnOH]^+^ complex displayed in Figure 7b is 13.3 kcal/mol more stable than its counterpart reported in Figure 7c.

**Figure 8:**
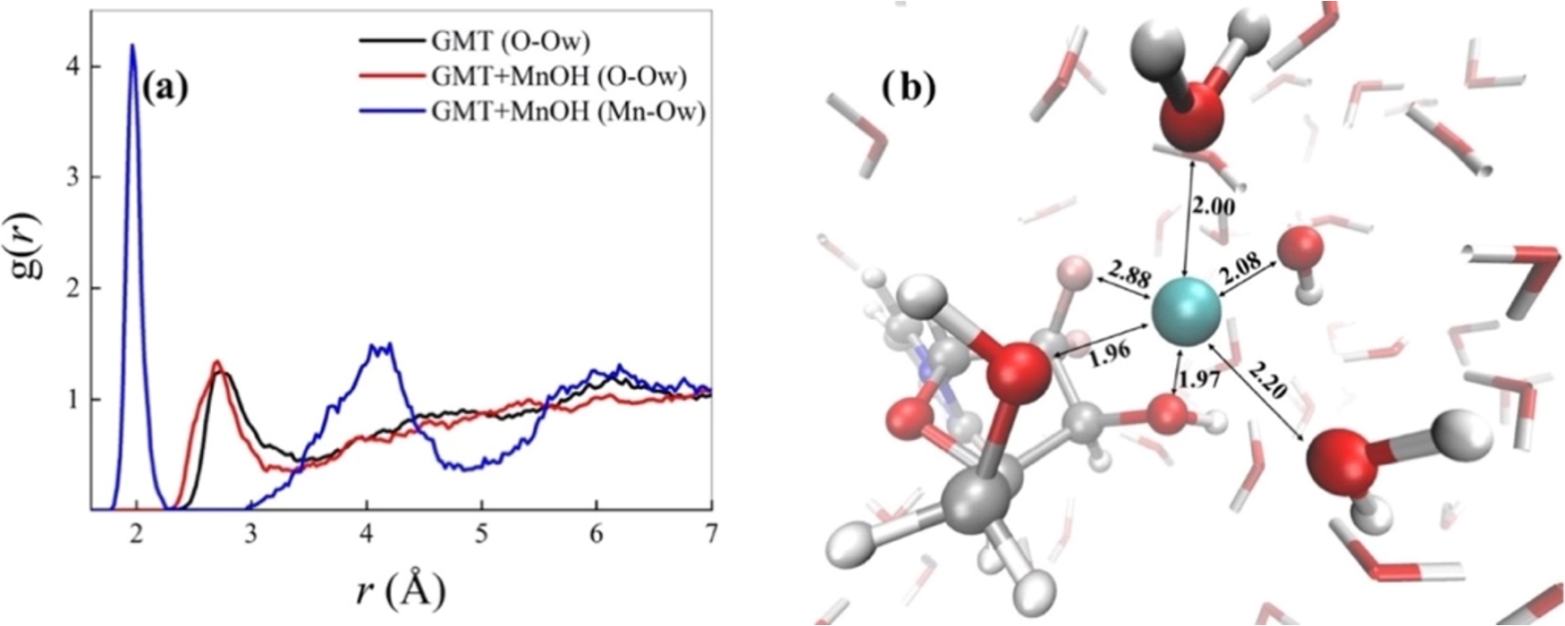
(a) Atomistic radial distribution functions between the oxygen atoms of GMT alone and the oxygen atoms of water (black curve), the oxygen atoms of GMT while chelating the [MnOH]^+^ and the oxygen atoms of water (red trace), and between the manganese atom in the latter complex and the oxygen atoms of the aqueous environment (blue line). (b) Typical solvation shell around the GMT+[MnOH]^+^ complex at the proximity of the manganese atom extracted from *ab initio* molecular dynamics simulations.

In light of the enhanced capability that [MnOH]^+^ exhibits in being chelated at the site1 of GMT and with the aim of exploring the role played by more realistic aqueous environments, we have performed an AIMD simulation of this GMT+[MnOH]^+^ structure surrounded by 143 explicitly treated H_2_O molecules at finite temperature. As a reference, also GMT solvated by 142 water species was simulated via AIMD. AIMD simulations of liquid systems can provide an efficient quantity that can be represented by the atomistic radial distribution functions g(r). These functions can highlight spatial correlations existing between given sets of atomic species. This way, to monitor the structure of the liquid environment around GMT, on the one hand, and of GMT+[MnOH]^+^, on the other hand, we determined the radial distribution function between all of the oxygen atoms of those species and the oxygen atoms of the surrounding aqueous system. As shown in Figure 8a, there exists a structuring of water molecules around the oxygen atoms of both GMT (black trace) and GMT+[MnOH]^+^ (red trace). Although GMT possesses 4 oxygen atoms in total, such structuring is essentially due to the two hydroxyl groups. In fact, the first peaks of those radial distribution functions are located at ≈2.8 Å, which corresponds to the typical length scale of hydrogen bonding in water under the simulated thermodynamic conditions. It is interesting to notice that when [MnOH]^+^ is chelated by GMT (red trace), the first peak of this radial distribution function falls at lower distances and gets narrower with respect to the case in which GMT alone is surrounded by water (black trace). This suggests that the first solvation shell around the GMT+[MnOH]^+^ complex is more structured and compact than the one shaping the local hydration of GMT.

By sitting on the manganese atom and by looking at the water environment, a very structurally enhanced situation emerges, as evidenced by the radial distribution function between Mn and the O atoms of water (O_w_) plotted in Figure 8a (blue trace). In fact, a sharp and high first peak characterizes the correlations between those atomic species whereas the complete depletion separating the latter peak from the second one witnesses the net separation of the first and second solvation shells around the chelated Mn moiety. Moreover, the location of the first peak of the Mn–O_w_ radial distribution function at ≈2.0 Å demonstrates that Mn^2+^ establishes very strong interactions with its own hydration shell. In fact, as depicted in Figure 8b, Mn is tightly bound not only to its own OH^–^ group and to the two chelating OH groups of GMT, but also to two additional H_2_O molecules stemming from the water environment and, to a lesser extent, to one of the fluorine atoms of GMT. In particular, the 3 OH^–^ groups and one of the H_2_O molecules span a quite compact equatorial plane whereas out-of-plane distances of the first solvation shell of Mn^2+^ appear to be statistically larger. Such an octahedral-like arrangement is in line with DFT computations on Mn^2+^-H_2_O (large enough) clusters.^52^ All evidence emerging from our AIMD simulations magnifies the necessity of invoking an explicit description of the water environment when dealing with GMT complexes of a similar nature to those here investigated, paving the way toward a more conspicuous usage of those computational techniques in this and related research fields. Our AIMD calculations showed that the presence of solvation H_2_O molecules leads to the formation of a stable [Mn(GMT)OH]^+^ species. Thus, metal GMT complexation will represent a good strategy for enhancing the physical stability of GMT nanoformulations, thereby preventing GMT leakage from carriers and consequent reductions in drug loading (%DL) over time, while maintaining the drug’s efficacy, as confirmed by the reported MTT assays.

## Conclusions

Our findings demonstrate that complex species of gemcitabine (GMT) with Mn^2+^ and Zn^2+^ (but not for Ca^2+^) under specific conditions achieve high formation percentages. These conditions are *t* = 45 °C, 1:1 molar ratio of metal-to-gemcitabine (M/GMT), and pH values of either 3 (commonly used to make liposomal formulations by the pH gradient method) or 7.4 (average physiological pH). Stabilization of [Mn(GMT)OH]^+^ complexes is elucidated at the molecular scale via Density Functional Theory calculations and spin-polarized *ab initio* molecular dynamics simulations. It turns out that the [MnOH]^+^ moiety is preferentially chelated by the two hydroxyl groups of GMT, whereas further stabilization of the complex is achieved by actively involving water molecules from the local hydration environment. The evidence of the significant complexing ability of GMT, mainly toward Mn^2+^, at *t* = 45 °C and pH = 3 and 7.4, and the involvement of the water molecules in conferring stability to the drug molecule will be the key factors for improving the drug loading in nanoformulations. Moreover, according to the *in vitro* results, GMT complexation with Mn^2+^ maintains the potency of the drug. The novelty of this research lies in its progression from speciation studies and computational calculations on drug-metal complexes to the concrete development of strategies for engineering new GMT liposomal and polymeric nanoparticle formulations. We are confident that our preliminary results will lead to novel GMT nanoformulations with enhanced quality attributes (better drug loading capacity and physical stability) and increased efficacy. The results reported in the current work hold the potential for achieving improved treatment outcomes, including enhanced efficacy and decreased toxicity, pivotal aspects paving the way for widespread usage of this potent therapeutic molecule.

## Supporting Information Available

Table S1: Hydrolysis Constants of Ca^2+^ at different temperatures and ionic strength values; Table S2: Hydrolysis Constants of Zn^2+^ at different temperatures and ionic strength values; Table S3: Hydrolysis Constants of Mn^2+^ at different temperatures and ionic strength values; Table S4: Cartesian components of the atomic species composing the gemcitabine molecular structures shown in Figure 7 of the main text optimized at the at the B3LYP/6-311++G(d,p) level of theory under CPCM water implicit solvation. Figure S1: ε vs wavelength of (a) Zn^2+^-GMT and (b) Mn^2+^–GMT at *t* = 45 °C, *I* = 0.1 mol L^–1^; Figure S2: UV spectra at selected pH of (a) Mn^2+^–GMT and (b) Zn^2+^–GMT at *t* = 45 °C, *I* = 0.1 mol L^–1^, *C*_GMT_ = 0.04 mmol L^–1^, *C*_M_ = 0.04 mmol L^–1^; Figure S3: ^1^H NMR spectra on solutions containing Zn^2+^–GMT at *C*_GMT_ = 5 mmol L^–1^ and *C*_Zn_ = 6 mmol L^–1^, *t* = 25 °C, *I* = 0.1 mol L^–1^ in NaCl, 2.70 ≤ pH ≤ 7.64; Figure S4. Comparison between chemical shift (δ) of (a) CH-5′ and (b) CH-6’ measured on ^1^H NMR spectra on solutions containing GMT and Zn^2+^–GMT at *C*_GMT_ = 5 mmol L^–1^ and *C*_Zn_ = 6 mmol L^–1^, *t* = 25 °C, *I* = 0.1 mol L^–1^ in NaCl, 2.72 ≤ pH ≤ 8.52 (PDF)

## Abbreviations

GMT: gemcitabine
FDA: Food and Drug Administration
WHO: World Health Organization
dFdCTP: GMT triphosphate
dFdCDP: GMT diphosphate
dFdU: 2′,2′-difluorodeoxyuridine
PK: pharmacokinetic
EE: entrapment efficacy
DL: drug loading
MTT: 3-(4, 5-dimethylthiazolyl-2)-2, 5-diphenyltetrazolium bromide
CPCM: conductive polarizable continuum model
DFT: Density Functional Theory
AIMD: *Ab Initio* Molecular Dynamics
