# Supplementary material for: Metal Complexation for the Rational Design of Gemcitabine Formulations in Cancer Therapy
Source: ACS Appl Mater Interfaces. 2024 Oct 8;16(42):56789–800. doi: 10.1021/acsami.4c12550 (PMC11503523; doi:10.1021/acsami.4c12550)
Supplement: Supplementary file 1 — am4c12550_si_001.pdf [file am4c12550_si_001.pdf]

# Supporting Information

# Metal complexation for the rational design of gemcitabine formulations in cancer therapy

Federica Carnamucio[1,*], Claudia Foti[2], Massimiliano Cordaro[2], Franz Saija[3], Giuseppe Cassone[3,*], Sandro R. P. da Rocha[1], Ottavia Giuffrè[2,*]

[1]*Department of Pharmaceutics and Center for Pharmaceutical Engineering and Sciences - School of Pharmacy, Virginia Commonwealth University, Richmond, VA 23284, United State*

[2] *Dipartimento di Scienze Chimiche, Biologiche, Farmaceutiche ed Ambientali, Università di Messina, Viale F. Stagno d'Alcontres 31, 98166 Messina, Italy*

[3] *Institute for Chemical-Physical Processes National Research Council of Italy, Viale Ferdinando Stagno d'Alcontres, 37, 98158 Messina, Italy*

[*]corresponding: FC (carnamuciof@vcu.edu); GC (giuseppe.cassone@ipcf.cnr.it); OG (ogiuffre@unime.it)

**Table S1.** Hydrolysis constants of $Ca^{2+}$ at different temperatures and ionic strength values.

| **Species** | ***t*** **/°C** | ***I*** **/ mol L$^{-1}$** | **logβ [1]** |
|---|---|---|---|
| $Ca^{2+} + H_2O \rightleftharpoons [CaOH]^{+} + H^{+}$ | 15 | 0.15 | -13.14 |
| | 25 | 0.15 | -12.87 |
| | 25 | 0.5 | -12.88 |
| | 25 | 1 | -12.81 |
| | 37 | 0.15 | -12.56 |
| | 45 | 0.15 | -12.6(5)[2] |

[1] Crea, F.; De Stefano, C., Milea, D., Pettignano, A., Sammartano, S. (2015). SALMO and S3M: A Saliva Model and a Single Saliva Salt Model for Equilibrium Studies, Bioinorg. Chem. Appl. 2015.
[2] Unplubished data.

**Table S2.** Hydrolysis constants of $Zn^{2+}$ at different temperatures and ionic strength values.

| Reaction | *t* /°C | *I* / mol L$^{-1}$ | logβ[1] |
|---|---|---|---|
| $Zn^{2+} + H_2O \rightleftharpoons [ZnOH]^+ + H^+$ | 15 | 0.15 | -9.5 |
| | 25 | 0.15 | -9.14 |
| | 25 | 0.5 | -9.15 |
| | 25 | 1 | -9.16 |
| | 37 | 0.15 | -8.78 |
| | 45 | 0.15 | -10.02[2] |
| $Zn^{2+} + 2H_2O \rightleftharpoons [Zn(OH)_2]^0 + 2H^+$ | 15 | 0.15 | -17.64 |
| | 25 | 0.15 | -17.10 |
| | 25 | 0.5 | -17.1 |
| | 25 | 1 | -17.22 |
| | 37 | 0.15 | -16.52 |
| | 45 | 0.15 | -16.52[2] |
| $Zn^{2+} + 3H_2O \rightleftharpoons [Zn(OH)_3]^- + 3H^+$ | 15 | 0.15 | -29.12 |
| | 25 | 0.15 | -28.4 |
| | 25 | 0.5 | -28.4 |
| | 25 | 1 | -28.47 |
| | 37 | 0.15 | -27.54 |
| | 45 | 0.15 | -27.54[2] |
| $Zn^{2+} + 4H_2O \rightleftharpoons [Zn(OH)_4]^{2-} + 4H^+$ | 15 | 0.15 | -41.67 |
| | 25 | 0.15 | -40.40 |
| | 25 | 0.5 | -40.85 |
| | 25 | 1 | -40.38 |
| | 37 | 0.15 | -39.47 |
| | 45 | 0.15 | -39.47[2] |
| $2Zn^{2+} + H_2O \rightleftharpoons [Zn_2(OH)]^{3+} + H^+$ | 15 | 0.15 | -9.27 |
| | 25 | 0.15 | -8.70 |
| | 25 | 0.5 | -8.89 |
| | 25 | 1 | -8.89 |
| | 37 | 0.15 | -8.54 |
| | 45 | 0.15 | -8.54[2] |
| $2Zn^{2+} + 6\,H_2O \rightleftharpoons [Zn_2(OH)_6]^{2-} + 6H^+$ | 15 | 0.15 | -58.91 |
| | 25 | 0.15 | -57.50 |
| | 25 | 0.5 | -57.53 |
| | 25 | 1 | -57.32 |
| | 37 | 0.15 | -55.9 |
| | 45 | 0.15 | -55.9[2] |

[1] F. Crea, G. Falcone, C. Foti, O. Giuffrè, S. Materazzi. Thermodynamic data for $Pb^{2+}$ and $Zn^{2+}$ sequestration by biologically important S-donor ligands, at different temperatures and ionic strengths, New J. Chem., 2014, 38, 3973.

[2] K. J. Powell, P. L. Brown, R. H. Byrne, T. Gajda, G. Hefter, A. Leuz, S. Sjöberg, H. Wanner, Chemical speciation of environmentally significant metals with inorganic ligands. Part 5: The $Zn^{2+}$ + $OH^–$, $Cl^-$, $CO_3^{2-}$, $SO_4^{2–}$, and $PO_4^{3–}$ systems (IUPAC Technical Report), Pure Appl. Chem., 2013, 85, 2249–2311.

**Table S3.** Hydrolysis constants of $Mn^{2+}$ at different temperatures and ionic strength values.

| **Reaction** | ***t* /°C** | ***I* / mol L$^{-1}$** | **logβ[1]** |
|---|---|---|---|
| $Mn^{2+} + H_2O \rightleftharpoons [MnOH]^{+} + H^{+}$ | 15 | 0.15 | -10.6 |
| | 25 | 0.15 | -10.46 |
| | 25 | 0.5 | -10.70 |
| | 25 | 1 | -10.95 |
| | 37 | 0.15 | -9.20 |
| | 45 | 0.1 | -9.66[2] |
| $2Mn^{2+} + 3H_2O \rightleftharpoons [Mn_2(OH)_3]^{-} + 3H^{+}$ | 15 | 0.15 | -26.35 |
| | 25 | 0.15 | -24.47 |
| | 25 | 0.5 | -24.69 |
| | 25 | 1 | -24.92 |
| | 37 | 0.15 | -23.51 |
| | 45 | 0.15 | -24.84[2] |

[1] C. Foti, O. Giuffrè. Interaction of Ampicillin and Amoxicillin with $Mn^{2+}$: A Speciation Study in Aqueous Solution. *Molecules*, 2020, *25*, 3110.

[2] Unpublished data.

**Table S4.** Cartesian components of the atomic species composing the gemcitabine molecular structures shown in Fig. 7 of the main text optimized at the at the B3LYP/6-311++G(d,p) level of theory under CPCM water implicit solvation.

**GMT**

| | X | Y | Z |
|---|---|---|---|
| N | -1.135780 | -0.149828 | 0.483570 |
| C | -2.040774 | -0.203497 | 1.509387 |
| C | -3.371839 | -0.229248 | 1.275122 |
| C | -3.785999 | -0.205182 | -0.097117 |
| N | -2.918044 | -0.164466 | -1.103343 |
| H | -1.630814 | -0.223674 | 2.510701 |
| H | -4.081883 | -0.271130 | 2.088047 |
| O | -0.736501 | -0.100685 | -1.763530 |
| C | -1.582652 | -0.137207 | -0.869090 |
| N | -5.096252 | -0.227095 | -0.400066 |
| H | -5.802926 | -0.255189 | 0.316358 |
| H | -5.384191 | -0.211832 | -1.366152 |
| C | 1.940284 | -0.955954 | -0.734941 |
| C | 1.954479 | 0.586184 | -0.878996 |
| C | 1.101855 | 1.072022 | 0.310024 |
| C | 0.293326 | -0.154015 | 0.804332 |
| H | 1.582037 | -1.397436 | -1.666622 |
| H | 0.347672 | -0.171846 | 1.893034 |
| O | 0.970346 | -1.277706 | 0.301313 |
| F | 0.307402 | 2.140486 | -0.005740 |
| F | 1.896779 | 1.497206 | 1.345396 |
| O | 3.264360 | 1.110392 | -0.902210 |
| H | 3.259642 | 1.929936 | -1.408394 |
| C | 3.256966 | -1.625569 | -0.358123 |
| H | 4.030807 | -1.314861 | -1.060127 |
| H | 3.124573 | -2.709827 | -0.450350 |
| O | 3.716015 | -1.290171 | 0.947452 |
| H | 3.054362 | -1.601631 | 1.576419 |
| H | 1.405997 | 0.858143 | -1.778739 |

**GMT+MnOH@site1**

| | X | Y | Z |
|---|---|---|---|
| N | 2.199392 | -0.011148 | 0.524085 |
| C | 3.093037 | -0.179605 | 1.549463 |
| C | 4.425221 | -0.186029 | 1.324765 |
| C | 4.854228 | -0.002821 | -0.031621 |
| N | 3.996725 | 0.169055 | -1.034208 |
| H | 2.672578 | -0.306819 | 2.538252 |
| H | 5.127223 | -0.320610 | 2.134532 |

| | | | |
|---|---|---|---|
| O | 1.820411 | 0.339686 | -1.695271 |
| C | 2.662544 | 0.178130 | -0.808936 |
| N | 6.165826 | 0.000113 | -0.322991 |
| H | 6.865893 | -0.122005 | 0.390258 |
| H | 6.463284 | 0.133518 | -1.277193 |
| C | -0.642433 | 1.311646 | -0.596645 |
| C | -0.909341 | -0.162174 | -0.988625 |
| C | -0.119178 | -0.978449 | 0.064735 |
| C | 0.772007 | 0.032888 | 0.831762 |
| H | -0.070813 | 1.786960 | -1.393879 |
| H | 0.689044 | -0.187045 | 1.895213 |
| O | 0.173017 | 1.285642 | 0.601353 |
| F | 0.578283 | -2.012381 | -0.479039 |
| F | -0.980507 | -1.542942 | 0.972007 |
| O | -2.304591 | -0.476425 | -1.004139 |
| H | -2.479123 | -1.248953 | -1.557645 |
| C | -1.850012 | 2.180306 | -0.306091 |
| H | -2.486138 | 2.255957 | -1.186328 |
| H | -1.516138 | 3.180862 | -0.028432 |
| O | -2.679777 | 1.657482 | 0.756992 |
| H | -2.231760 | 1.745287 | 1.608405 |
| H | -0.485848 | -0.369869 | -1.966879 |
| Mn | -3.961057 | 0.033511 | 0.318252 |
| O | -5.058273 | -1.305149 | -0.419477 |
| H | -5.758089 | -1.666532 | 0.133971 |

**GMT+MnOH@site2**

| | X | Y | Z |
|---|---|---|---|
| N | -0.260203 | -0.318647 | 0.532309 |
| C | 0.559206 | -0.696567 | 1.551577 |
| C | 1.884891 | -0.917024 | 1.346856 |
| C | 2.345799 | -0.724745 | 0.021101 |
| N | 1.587210 | -0.348703 | -0.976085 |
| H | 0.099622 | -0.809572 | 2.524408 |
| H | 2.536300 | -1.211803 | 2.155791 |
| O | -0.513046 | 0.203992 | -1.680094 |
| C | 0.248214 | -0.129909 | -0.781632 |
| N | 3.721626 | -0.906259 | -0.269847 |
| H | 4.116735 | -1.707402 | 0.220402 |
| H | 3.855562 | -1.024888 | -1.272451 |
| C | -3.068986 | 1.278023 | -0.564938 |
| C | -3.351271 | -0.177831 | -1.011562 |
| C | -2.666936 | -1.035743 | 0.072324 |
| C | -1.689252 | -0.093853 | 0.821147 |

| | | | |
|---|---|---|---|
| H | -2.591649 | 1.817562 | -1.384452 |
| H | -1.798911 | -0.271407 | 1.891160 |
| O | -2.104404 | 1.207708 | 0.525666 |
| F | -2.044167 | -2.140166 | -0.442781 |
| F | -3.576159 | -1.510314 | 0.979926 |
| O | -4.729312 | -0.432930 | -1.162065 |
| H | -4.846459 | -1.131632 | -1.815013 |
| C | -4.261487 | 2.093716 | -0.079030 |
| H | -5.039895 | 2.073323 | -0.841872 |
| H | -3.933597 | 3.131492 | 0.049212 |
| O | -4.840831 | 1.601288 | 1.124620 |
| H | -4.184614 | 1.694348 | 1.825093 |
| H | -2.811714 | -0.370236 | -1.937718 |
| Mn | 5.105583 | 0.756972 | 0.307625 |
| O | 6.598384 | 0.602033 | -0.826014 |
| H | 6.914154 | -0.277628 | -1.056073 |

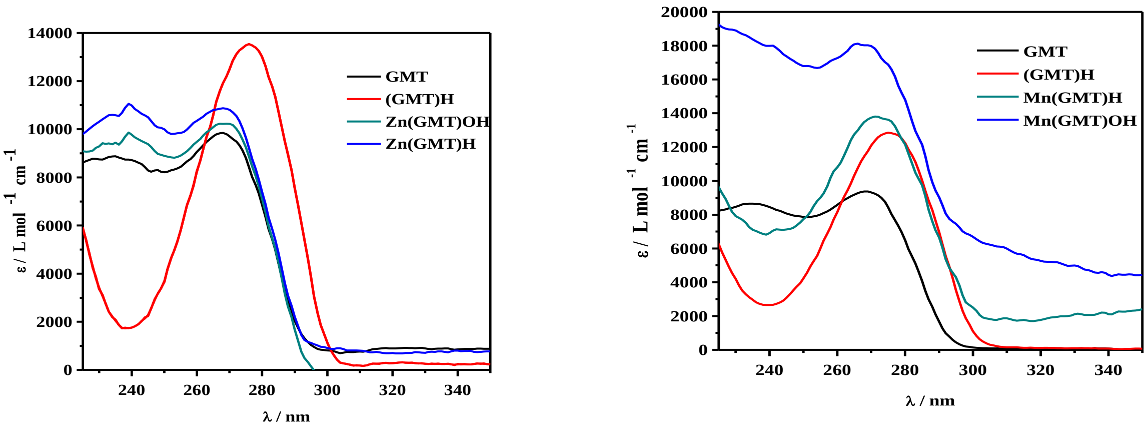


**(a)** **(b)**

**Figure S1.** ε vs. λ of **a)** and $Zn^{2+}$-GMT **b)** $Mn^{2+}$-GMT species at $t$ = 45°C, $I$ = 0.1 mol $L^{-1}$.

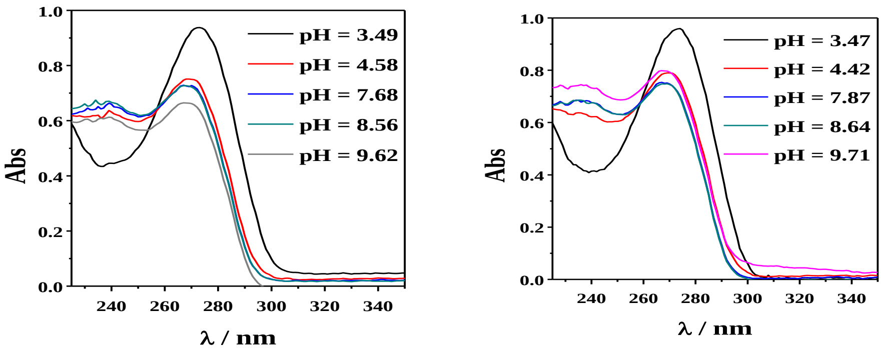


(**a**) (**b**)

**Figure S2.** UV spectra at selected pH of a) $Mn^{2+}$-GMT and b) $Zn^{2+}$-GMT at $t$ = 45°C, $I$ = 0.1 mol $L^{-1}$, $C_{GMT}$ = 0.04 mmol $L^{-1}$, $C_M$ = 0.04 mmol $L^{-1}$.

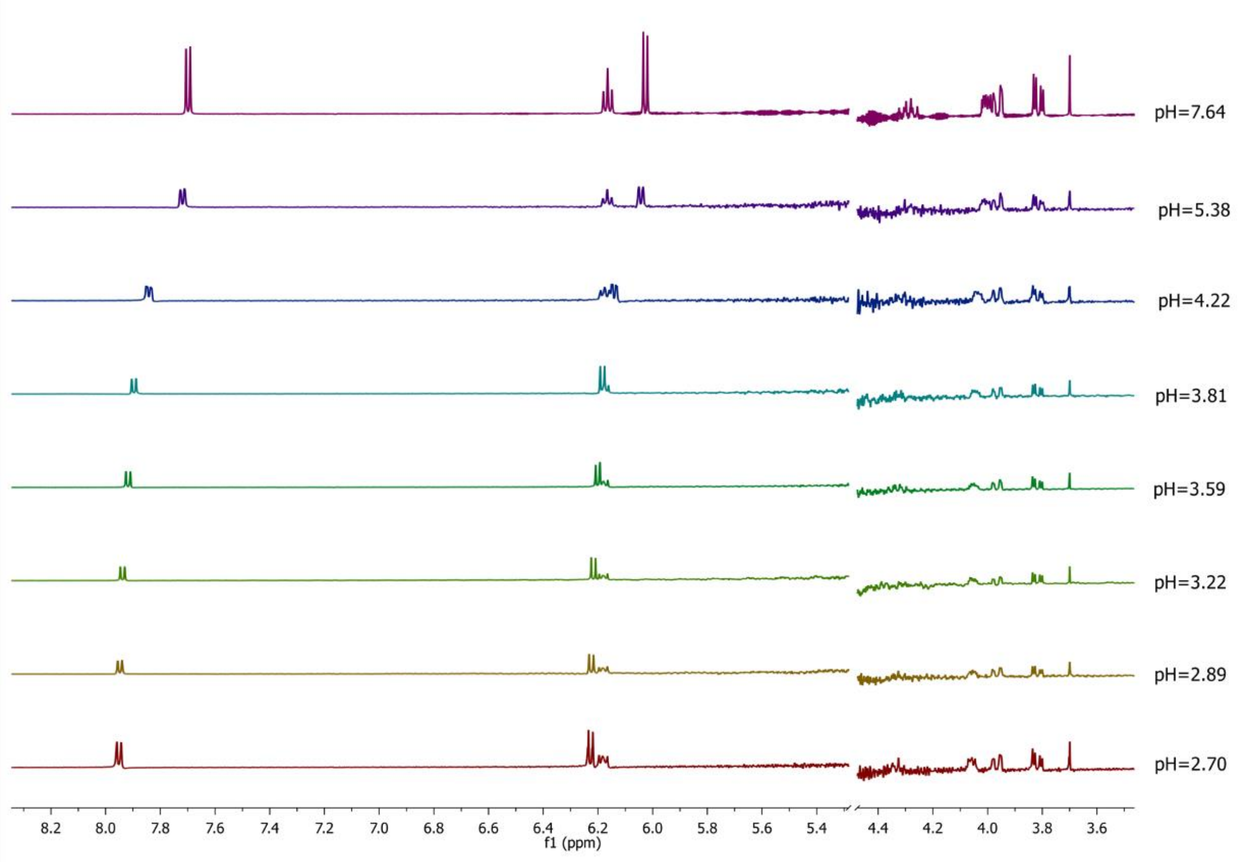


**Figure S3.** $^1$H NMR spectra on solutions containing $Zn^{2+}$-GMT at $C_{GMT}$ = 5 mmol $L^{-1}$ and $C_{Zn}$ = 6 mmol $L^{-1}$, $t$ = 25°C, $I$ = 0.1 mol $L^{-1}$ in NaCl, 2.70 ≤ pH ≤ 7.64.

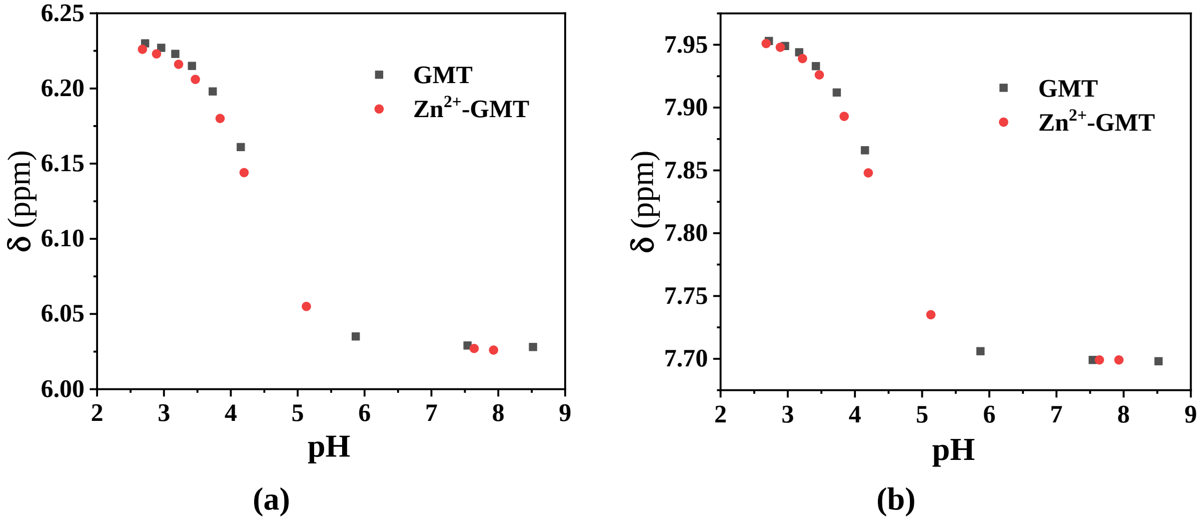


**Figure S4.** Comparison between chemical shift (δ) of a) CH-5' and b) CH-6' measured on $^1$H NMR spectra on solutions containing GMT and $Zn^{2+}$-GMT at $C_{GMT}$ = 5 mmol $L^{-1}$ and $C_{Zn}$ = 6 mmol $L^{-1}$, $t$ = 25°C, $I$ = 0.1 mol $L^{-1}$ in NaCl, 2.72 ≤ pH ≤ 8.52.
